# Analysis for global characteristics of Lyapunov exponents in vehicle plane motion system

**DOI:** 10.1038/s41598-022-13411-x

**Published:** 2022-06-03

**Authors:** Fanyu Meng, Shuming Shi, Boshi Zhang, Minghui Bai, Nan Lin

**Affiliations:** College of Transportation, Jilin University, Changchun, China

**Keywords:** Nonlinear phenomena, Mechanical engineering

## Abstract

In the field of vehicle system dynamics, it is significant to propose an appropriate quantitative indicator for system stability or dynamics characteristics. Lyapunov exponents method is an excellent quantitative indicator for analysing nonlinear system characteristics. It was used to studied the stable region of nonlinear vehicle plane motion system. However, the effect of Lyapunov exponents method in revealing the global dynamics characteristics has not been fully studied. Aiming at this key problem, this paper analyses the global characteristics of Lyapunov exponents with different degrees of freedom nonlinear models. The results show that Lyapunov exponents can well reflect the global layer characteristics for vehicle system. However, the value characteristic under different DOF models is not unified. The research and analysis in this paper supplement the quantitative analysis theory for vehicle system dynamics.

## Introduction

As the land vehicle becomes more and more intelligent, the power performance becomes better, and the traffic environment becomes more complex. Researchers have higher and higher requirements for nonlinear and high-DOF vehicle system dynamics methods. The most classical theory of vehicle dynamics is based on the linear 2-DOF vehicle model. The lateral stability is analysed by characteristic roots of ordinary differential equations^1–3^. Due to the limitation of linearization, classical theory cannot accurately describe the global characteristics (stable region + unstable region characteristics) for high-speed nonlinear vehicle model. Therefore, in the past 30 years, many researchers have carried out numerous researches on the dynamic characteristics for nonlinear vehicle model.

The phase plane method proposed by Inagaki et al.^4^, based on nonlinear tire model and 2-DOF vehicle model, provided a theoretical basis for vehicle dynamics analysis and control. For example, Toyota took the stability region obtained by this method as the target region to maintain vehicle stable^5^. Ono et al.^6^ also used the phase plane method to analyse the dynamic characteristics of 2-DOF nonlinear vehicle model, and showed that the unstable motion under high speed and large front wheel steering angle condition is essentially caused by the saddle-node bifurcation of the equilibrium point of the system. Catino^7^ and Nguyen^8^ researched the bifurcation phenomenon caused by front wheel steering angle in 2-DOF model. Shen et al.^9^ used the joint-point locus approach to analyze the dynamic stability of a 2-DOF nonlinear vehicle model with a simplified tire magic formula. Horiuchi et al.^10,11^ proposed a method for analysing vehicle stability during acceleration and braking using constrained bifurcation and continuation methods, then proposed a new systematic evaluation approach for vehicle maneuverability based on controllability region computations. Ko et al.^12,13^ used topology theory to solve the stable region of a 3-DOF (longitudinal motion, lateral motion and yaw motion) nonlinear model by using trajectory reversal method. Shi et al.^14^ use phase space to analyse the characteristics of 3-DOF model and show the vehicle motion characteristics under the extreme tire conditions.

Phase plane method is one of the most classical methods in nonlinear vehicle dynamics. By observing the evolution of phase trajectories, this method can intuitively and clearly obtain the equilibrium point and bifurcation characteristics, as well as the change trend of the size and shape of stable region under different parameters, which is easy to be popularized in engineering and control fields. Although this method can find the stable region boundary or bifurcation point of the system, state points in phase space are directly labelled as "stable" or "unstable", then their colourful personalities are hidden by these "black or white" labels.

In addition to the phase plane method, Lyapunov function method^15^ and Lyapunov exponents method^16^ are also significant methods for analysing vehicle system dynamic characteristics. Lyapunov function method is difficult to be applied to high-DOF vehicle system due to its high dependence on mathematical experience. As for the Lyapunov exponents method, Christine Wu et al.^16^ first studied its application in stability analysis for a 2-DOF model (whose tire force is expressed as a cubic polynomial). According to their research, the largest Lyapunov exponent is an indicator of the convergence rate that characterizes the vehicle system's ability to return to a stable driving state after being disturbed. Shi et al.^17^ researched the Lyapunov exponent characteristics in higher-DOF model, and showed that in the stable region of the 3-DOF or 5-DOF model, the Lyapunov exponent at different initial state points are different. For Lyapunov exponent. these researches are aiming at the stable region, which can provide quantitative calculation results and supplement the conclusion of phase plane method and other methods. In addition, Meng et al.^18^ researched the dynamics characteristics in vehicle global region based on dissipation of energy, which is a quantitative indicator can reveal vehicle global layer characteristics. Researches about Lyapunov exponents in other engineering fields is also very important. For example, A. Tamer and P. Masarati researched Lyapunov exponents in nonlinear rotorcraft stability problems^19^ and suggested the formulation of Lyapunov exponent stability^20^.They also presented the use of Lyapunov exponents in the design of nonlinear systems^21^ and studied the extraction of Lyapunov exponents from multibody dynamic models^22^.

Based on the above research results, it can be seen that the current researches of handling stability based on nonlinear vehicle model mainly focused on bifurcation, equilibrium point and finding the stable region for low-DOF (2-DOF or 3-DOF) models. Classical methods, such as phase plane method or Lyapunov function method, mostly can get the qualitative conclusions and lack definite quantitative indicators. Therefore, it is necessary to further research the quantitative Lyapunov exponents characteristics for high-DOF models in global regions.

In order to solve the existing problems, the differential equations of 2, 3 and 5-DOF vehicle models are given in “2-DOF,3-DOF,5-DOF vehicle model” vehicle model. The concept and calculation method of Lyapunov exponent are briefly introduced in “Concept and calculation of Lyapunov exponent”. The global Lyapunov exponents characteristics of 2, 3 and 5-DOF vehicle models are analysed in “Analysis for global characteristics of 2, 3, 5-DOF models”. The conclusions are given in “Conclusions and future work”.

## 2-DOF,3-DOF,5-DOF vehicle model

As introduced in the section “Introduction”, the 2-DOF vehicle model is the most classical model for vehicle dynamic analysis. This model assumes that the longitudinal velocity of the vehicle is fixed, and ignores the pitch, roll and load transfer of the vehicle. Only the lateral motion along the Y-axis and the yaw motion around the Z-axis are considered (using the vehicle ISO coordinate system). The specific equation is as follows:

1 $$\left\{\begin{array}{c}{\dot{v}}_{y}=-{v}_{x}\omega +\frac{{F}_{sf}\mathrm{cos}{\delta }_{f}+{F}_{sr}}{m}\\ \dot{\omega }=\frac{{F}_{sf}\mathrm{cos}{\delta }_{f}\cdot {l}_{f}-{F}_{sr}\cdot {l}_{r}}{{I}_{z}}\end{array}\right.$$

**Figure 1:**
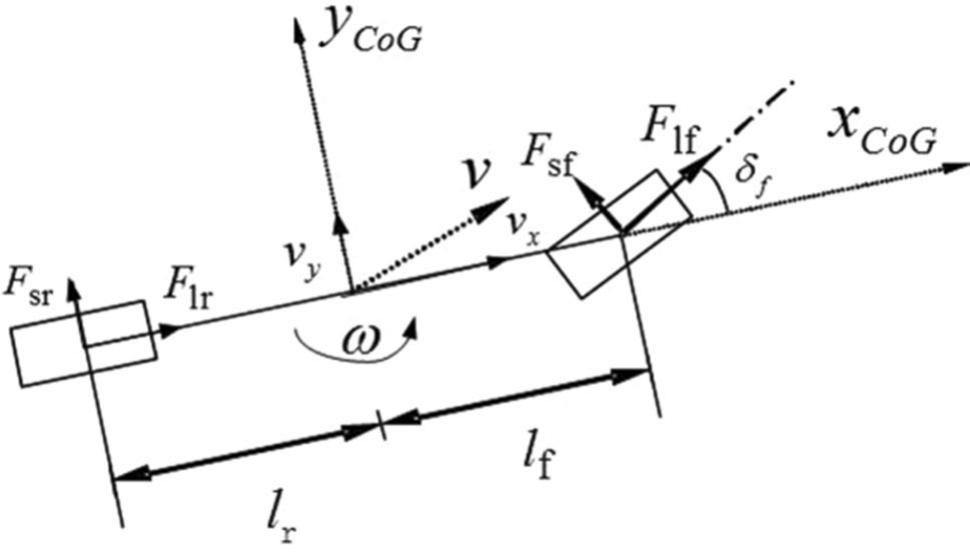
Vehicle single-track model.

In this equation, m ($kg$) is the vehicle mass;${I}_{z}(\mathrm{kg}\cdot {\mathrm{m}}^{2})$ is the moment of inertia of the vehicle around the Z-axis. ${v}_{x}(\mathrm{m}/\mathrm{s}) ,{v}_{y}(\mathrm{m}/\mathrm{s})$ are longitudinal and lateral velocity at the CG, respectively.$\omega (\mathrm{rad}/\mathrm{s})$ is yaw rate of vehicle.${\delta }_{f}(\mathrm{rad})$ is front wheel steering angle.${F}_{sf}(N)$ and ${F}_{sr}(N)$ are lateral force at front and rear axles (combining force of two tires), respectively. ${l}_{f}(m)$ and ${l}_{r}(m)$ are distance from CG to front and rear axles, respectively. Figure 1 is the vehicle single-track model used in this paper. The 2-DOF model is classic because it can cover the basic vehicle structure parameters and tire parameters without losing the essence of vehicle dynamics. In addition, because of its simple equation, it is convenient to calculate analytical solution or analyse. This is why the high-DOF model is often simplified to 2-DOF model before mathematical analysis in many researches. The 2-DOF model nonlinearity is mainly from ${v}_{x}\omega$, ${F}_{sf}\mathrm{cos}{\delta }_{f}$.

The classical 2-DOF model assumes that the longitudinal velocity is unchanged. Therefore, the coupling characteristic between longitudinal and lateral motion of the vehicle cannot be not considered in 2-DOF, which means the lateral velocity cannot influence the longitudinal velocity and the 2-DOF model cannot reflect the real vehicle motion. It is necessary to consider longitudinal velocity change on the basis of the 2-DOF model^14^. Therefore, the 3-DOF vehicle plane motion system model can be obtained from 2-DOF model, including lateral velocity, yaw rate and longitudinal velocity (as shown in Eq. (2)).

2 $$\left\{\begin{array}{c}\dot{{v}_{y}}=-{v}_{x}\omega +\frac{{F}_{sf}\mathit{cos}{\delta }_{f}+{F}_{sr}}{m}\\ \dot{\omega }=\frac{{F}_{sf}\mathit{cos}{\delta }_{f}\cdot {l}_{f}-{F}_{sr}\cdot {l}_{r}}{{I}_{z}}\\ \dot{{v}_{x}}={v}_{y}\omega -\frac{{F}_{sf}\mathit{sin}{\delta }_{f}}{m}\end{array}\right.$$

As shown in Eq. (2), the $\dot{{v}_{y}}$ is calculated by the $-{v}_{x}\omega$. On the basis of the 2-DOF model, the assumption about fixed ${v}_{x}$ is removed and the longitudinal-lateral velocity coupling is introduced. In theory, the 3-DOF model can more realistically reflect the vehicle motion phenomenon both in stable and unstable region. 3-DOF model introduces new nonlinearity, such as ${v}_{y}\omega$ and ${F}_{sf}\mathit{sin}{\delta }_{f}$.

Based on the 3-DOF model, the 5-DOF vehicle plane motion model can be constructed by introducing the rotation of front and rear wheels, in which the driving torque and tire longitudinal force are considered. The 5-DOF model includes the longitudinal motion along the X-axis, the lateral motion along the Y-axis, the yaw motion around the Z-axis, and the rotational motion of the front and rear wheels around the respective axles. The differential equation of 5-DOF model is:

3 $$\left\{\begin{array}{c}{\dot{v}}_{y}=-{v}_{x}\omega +\frac{{F}_{lf}\mathrm{sin}{\delta }_{f}+{F}_{sf}\mathrm{cos}{\delta }_{f}+{F}_{sr}-\mathrm{sgn}\left({v}_{y}\right)\cdot {C}_{y}{A}_{y}\frac{{\rho }_{air}}{2}{v}_{y}^{2}}{m}\\ \dot{\omega }=\frac{\left({F}_{lf}\mathrm{sin}{\delta }_{f}+{F}_{sf}\mathrm{cos}{\delta }_{f}\right){l}_{f}-{F}_{sr}{l}_{r}}{{I}_{z}}\\ {\dot{v}}_{x}={v}_{y}\omega +\frac{{F}_{lf}\mathrm{cos}{\delta }_{f}-{F}_{sf}\mathrm{sin}{\delta }_{f}+{F}_{lr}-\mathrm{sgn}\left({v}_{x}\right)\cdot {C}_{x}{A}_{x}\frac{{\rho }_{air}}{2}{v}_{x}^{2}}{m}\\ {\dot{\omega }}_{f}=\frac{{T}_{df}-{R}_{e}\cdot {F}_{lf}}{J}\\ {\dot{\omega }}_{r}=\frac{{T}_{dr}-{R}_{e}\cdot {F}_{lr}}{J}\end{array}\right.$$

where,${\omega }_{f},{\omega }_{r}(rad/s)$ are front wheel angular velocity and rear wheel angular velocity respectively; $J(kg\cdot {m}^{2})$ is the moment of inertia of wheel rotation; ${C}_{x}$ and ${C}_{y}$ are the longitudinal and lateral coefficient of air resistance respectively. ${A}_{x}({m}^{2})$ and ${A}_{y}({m}^{2})$ are the longitudinal and lateral windward area respectively.${\rho }_{air}\left(kg/{m}^{3}\right)$ is air density; ${T}_{df}(N\cdot m)$ and ${T}_{dr}(N\cdot m)$ are the driving torque on front and rear wheels respectively. ${R}_{e}(m)$ is the wheel radius. ${F}_{lf }\left(N\right)$ and ${F}_{lr}\left(N\right)$ are the longitudinal forces of front and rear wheels respectively. It can be seen that the nonlinearity of the 5-DOF model is more complex.

The vehicle structural parameters are selected according to paper^6^, as shown in Table 1.

**Table 1:** Structural parameters of vehicle.

Symbol	Parameter values	Symbol	Parameter values
$m\left(kg\right)$	1500	${C}_{y}$	0.4
${I}_{z}\left(kg\cdot {m}^{2}\right)$	3000	${A}_{x}\left({m}^{2}\right)$	1.7
${l}_{f}\left(m\right)$	1.2	${A}_{y}\left({m}^{2}\right)$	3.5
${l}_{r}\left(m\right)$	1.3	$J\left(kg\cdot {m}^{2}\right)$	2.0
${C}_{x}$	0.3	${R}_{e}\left(m\right)$	0.224
		${\rho }_{air}\left(kg/{m}^{3}\right)$	1.2258

In this paper, the magic formula^23,24^ is used to calculate the tire force. The formula can accurately express the mechanical characteristics of tires by a set of calculation equations, which has a high fitting accuracy, especially in the non-linear region. The calculation equation is as follows:

4 $$Y=D\mathrm{sin}\left(C\mathrm{arctan}\left(Bx-E\left(Bx-\mathrm{arctan}Bx\right)\right)\right)$$

In the Eq. (4), B is the stiffness factor; C is the shape factor; D is the peak factor; E is the curvature factor; x is one of the tire slip rate (${k}_{f}$,${k}_{r}$ ) or sideslip angle (${\alpha }_{f}$,${\alpha }_{r}$); Y is one of the steady-state longitudinal force (${F}_{lf0}$,${F}_{lr0}$) or steady-state lateral force (${F}_{sf0}$,${F}_{sr0}$) of the tire. Equation (4) is also one of the sources of nonlinearity in the vehicle system. The calculation equation of these variables will be introduced following.

According to the paper^6^, this paper uses two sets of tire force parameters under high and low adhesion road conditions for simulation analysis, as shown in Tables 2 and 3:

**Table 2:** Tire force parameters under high adhesion road.

Force	Wheel	B	C	D	E
Longitudinal force parameter	Front	6.7651	1.3	6436.8	− 1.999
	rear	9.0051	1.3	5430	− 1.7908
Lateral force parameters	Front	6.7651	1.3	6436.8	− 1.999
	rear	9.0051	1.3	5430	− 1.7908

**Table 3:** Tire force parameters under low adhesion road.

Force	Wheel	B	C	D	E
Longitudinal force parameter	Front	11.275	1.56	2574.8	0.4109
	rear	18.631	1.56	1749.6	0.4108
Lateral force parameters	Front	11.275	1.56	2574.7	− 1.999
	rear	18.631	1.56	1749.7	− 1.7908

The tire slip rate model suitable for all working conditions^25^ is used to calculate the tire slip rate of the front and rear wheels, as shown in Eq. (5):

5 $$\left\{\begin{array}{c}{k}_{f}=\frac{{\omega }_{f}\cdot {R}_{e}-{v}_{xf}}{max(\left|{\omega }_{f}{R}_{e}\right|,\left|{v}_{xf}\right|)}\\ {k}_{r}=\frac{{\omega }_{r}\cdot {R}_{e}-{v}_{xr}}{max(\left|{\omega }_{r}{R}_{e}\right|,\left|{v}_{xr}\right|)}\end{array}\right.$$

In this equation, ${k}_{f}$ and ${k}_{r}$ are the slip rate of the front and rear wheels; ${v}_{xf}(m/s)$ and ${v}_{xr}(m/s)$ are the longitudinal velocity of the front and rear wheels respectively. The calculation equation for ${v}_{xf}$ and ${v}_{xr}$ is:

6 $$\left\{\begin{array}{c}{v}_{xf}={v}_{x}\cdot \mathrm{cos}{\delta }_{f}+\left({v}_{y}+{l}_{f}\cdot \omega \right)\cdot \mathrm{sin}{\delta }_{f}\\ {v}_{xr}={v}_{x}\end{array}\right.$$

About the calculation of tire sideslip angle, the unified calculation model of tire side slip angle^23,25^ is adopted, and the equation is follows:

7 $$\left\{\begin{array}{c}{\alpha }_{f}=\left({\delta }_{f}-\mathrm{arctan}\left(\frac{{v}_{y}+\omega \cdot {l}_{f}}{{v}_{x}}\right)\right)\cdot sgn\left({v}_{xf}\right)\\ {\alpha }_{r}=\left(-\mathrm{arctan}\left(\frac{{v}_{y}-\omega \cdot {l}_{r}}{{v}_{x}}\right)\right)\cdot sgn\left({v}_{xr}\right)\end{array}\right.$$

where, ${\alpha }_{f}$ and ${\alpha }_{r}$ are the sideslip angle of the front and rear wheels. In order to fully describe the dynamics characteristics of the tire force lateral-longitudinal coupling, it is necessary to fully consider the side slip characteristics of the tire, which is the “friction ellipse” relationship between the tire and the ground. The specific calculation method^23^ is in Eq. (8):

8 $$\left\{\begin{array}{c}{F}_{lf}={F}_{lf0}\cdot {G}_{x}\\ {F}_{lr}={F}_{lr0}\cdot {G}_{x}\\ {G}_{x}=\mathrm{cos}\left[\mathrm{arctan}\left\{{B}_{g,x}\left(\alpha \right)\cdot \alpha \right\}\right]\\ {B}_{g,x}\left(\alpha \right)={r}_{x,1}\mathrm{cos}\left[\mathrm{arctan}\left({r}_{x,2}\cdot k\right)\right]\\ {F}_{sf}={F}_{sf0}\cdot {G}_{y}\\ {F}_{sr}={F}_{sr0}\cdot {G}_{y}\\ {G}_{y}=\mathrm{cos}\left[\mathrm{arctan}\left\{{B}_{g,x}\left(k\right)\cdot k\right\}\right]\\ {B}_{g,y}\left(k\right)={r}_{y,1}\mathrm{cos}\left[\mathrm{arctan}\left({r}_{y,2}\cdot \alpha \right)\right]\end{array}\right.$$

In this equation, ${G}_{x}$, ${G}_{y}$ are tire force mixed side slip correction functions; ${r}_{x,1}$, ${r}_{x,2}$, ${r}_{y,1}$, ${r}_{y,2}$ are tire mixed side slip correction coefficients (The values are shown in Table 4); ${F}_{lf0}$ and ${F}_{lr0}$ are the longitudinal force of the front and rear wheel in steady state; ${F}_{sf0}$ and ${F}_{sr0}$ are the lateral force of the front and rear wheel in the steady state; $\alpha$ and $k$ are the tire side slip angle and tire slip rate, respectively.

**Table 4:** Tire combined slip coefficients.

Longitudinal slip coefficient		Lateral slip coefficient	
${r}_{x,1}$	${r}_{x,2}$	${r}_{y,1}$	${r}_{y,2}$
35	40	40	35

## Concept and calculation of Lyapunov exponent

The concept of Lyapunov exponents was first proposed by Lyapunov in order to study the stability of nonstationary solutions of ordinary differential equations^26^. Lyapunov exponents can reflect the stability of states or trajectories, which reveals whether two adjacent trajectories are close to each other or separate over time. Because of this property, in the field of nonlinear dynamics, Lyapunov exponents are often used to judge whether the system has chaotic phenomenon. In chaotic systems, the sensitive dependence of chaotic motion on initial states makes phase trajectories inevitably separate. Using the Lyapunov exponents can quantitatively reflect the stability of phase trajectories, which can indicate the convergence characteristics of nonlinear system.

Considering a continuous system with n-dimensional states, the Lyapunov exponents can detect the long-term evolutionary behaviour of an n-dimensional sphere with infinitely small initial conditions. Due to dynamic manifold changes, this n-dimensional sphere may transform into an n-dimensional ellipsoid. Given $p$ as the radius of this sphere, the length of the *i-*th axis of the ellipsoid is ${p}_{i}(t)$. The average length change rate of the ellipsoid principal axis in infinite time is called Lyapunov exponent, and its calculation formula can be expressed as:

9 $${\lambda }_{i}=\underset{t\to \infty }{\mathrm{lim}}\frac{1}{t}{\mathrm{log}}_{2}\frac{{p}_{i}(t)}{{p}_{i}(0)}$$

Since each principal axis can define a Lyapunov exponent, an n-dimensional system has n Lyapunov exponents ${\lambda }_{i}$ in descending order. Note that the direction of the ellipsoid principal axis changes over time as it evolves, so it is not possible to define the direction associated with a given exponent. The relationship needs to be further considered between the evolution of the ellipsoid and the Lyapunov exponent. When a certain Lyapunov exponent is negative, it indicates that the ellipsoid is average contracted in a varying principal axis direction, and also reflects that the adjacent phase trajectories are convergent in a varying direction. Similarly, when a Lyapunov exponent is positive, it means that the ellipsoid is average divergent in a varying direction, and the adjacent phase trajectories are divergent in a varying direction. The negative Lyapunov exponent represents the stable and convergent motion state; the positive Lyapunov exponent represents unstable and divergent motion state; when the Lyapunov exponent is 0, it represents that the solution is neither exponentially convergent nor exponentially divergent. When analyzing the dynamical characteristics of a system, Lyapunov exponents has two important indicators: the largest Lyapunov exponent and the sum of Lyapunov exponents. The largest Lyapunov exponent reflects the convergent speed in the largest convergence direction of the phase trajectories. The sum of the Lyapunov exponents is the time-averaged divergence of the phase space velocity^27^.

**Figure 2:**
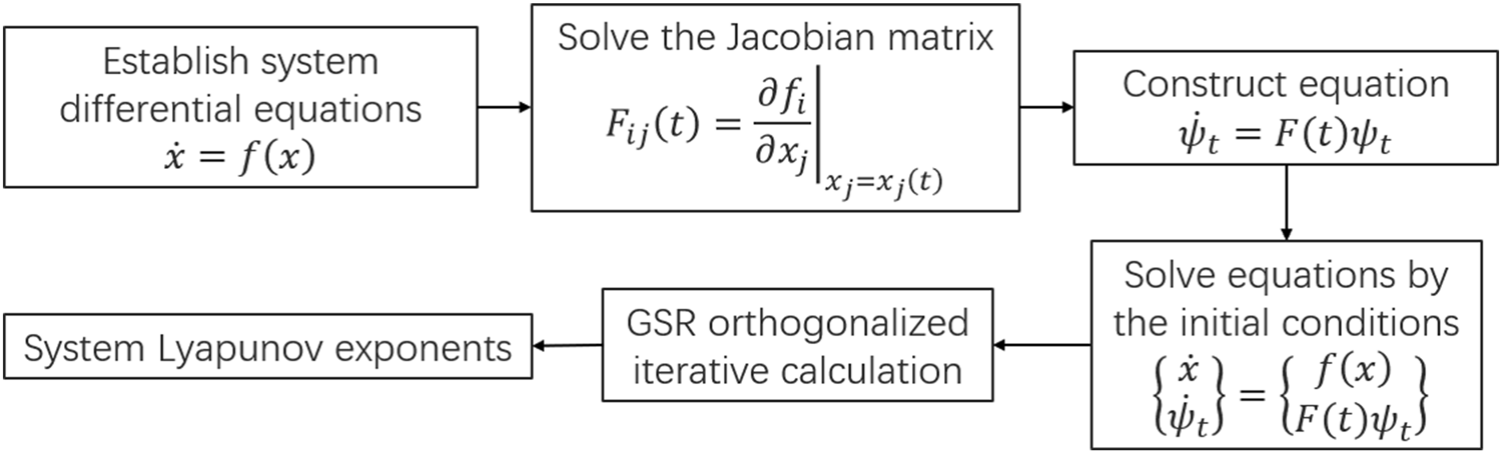
Calculation process of Lyapunov exponents.

Since Lyapunov exponent was proposed, many researchers have researched it and proposed new numerical calculation method. However, the results using different numerical calculation methods are slightly different, thus the numerical calculation method should be selected according to application scenes^28^. Because vehicle system has strong nonlinear characteristics in unstable region, this paper selects the orthodontic method summarized by Wolf^27^ to calculate the Lyapunov exponents. This method is one of the most classical methods to solve Lyapunov exponent of ordinary differential equation and can be used in most nonlinear systems, which is adopted by Wu et al.^16^ and Shi et al.^17^ to analyse vehicle system. Therefore, Wolf’s method is also used in this paper. It is worth noting that the numerical calculation method of Lyapunov exponents in engineering application is to estimate the theoretical value, rather than to calculate the exact solution. The calculation process of Wolf’s method^27^ is shown in Fig. 2.

**Figure 3:**
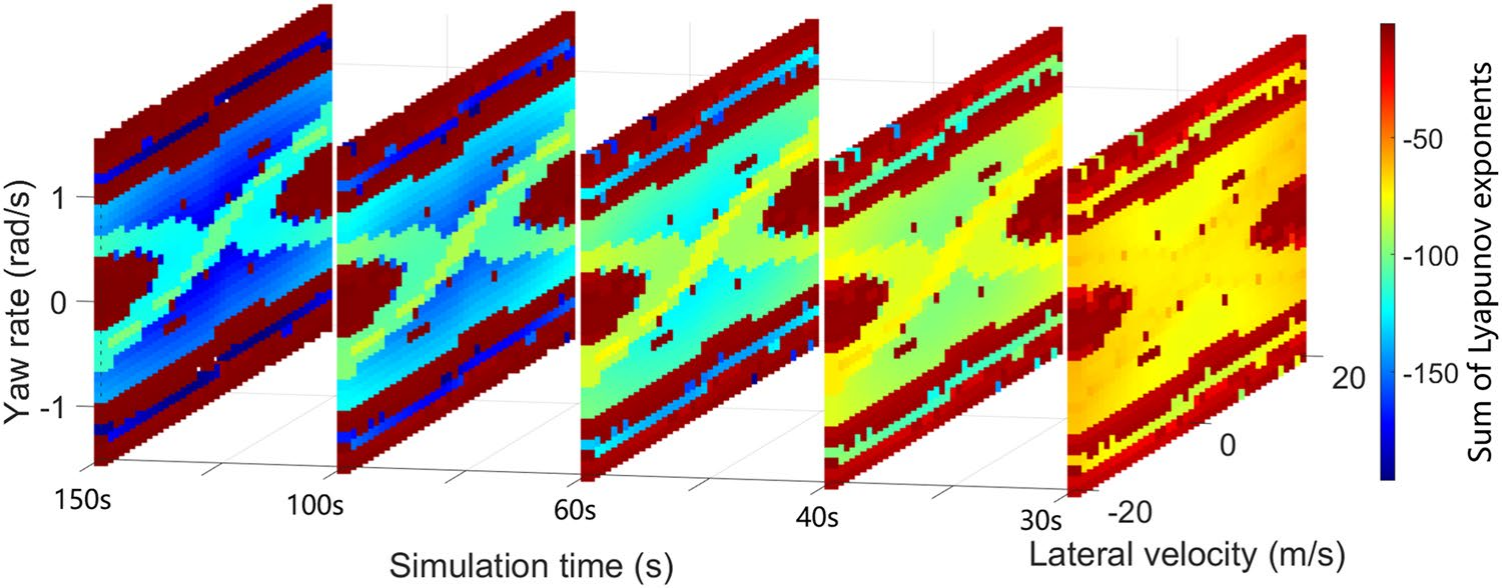
Sum of Lyapunov exponents at different time.

**Figure 4:**
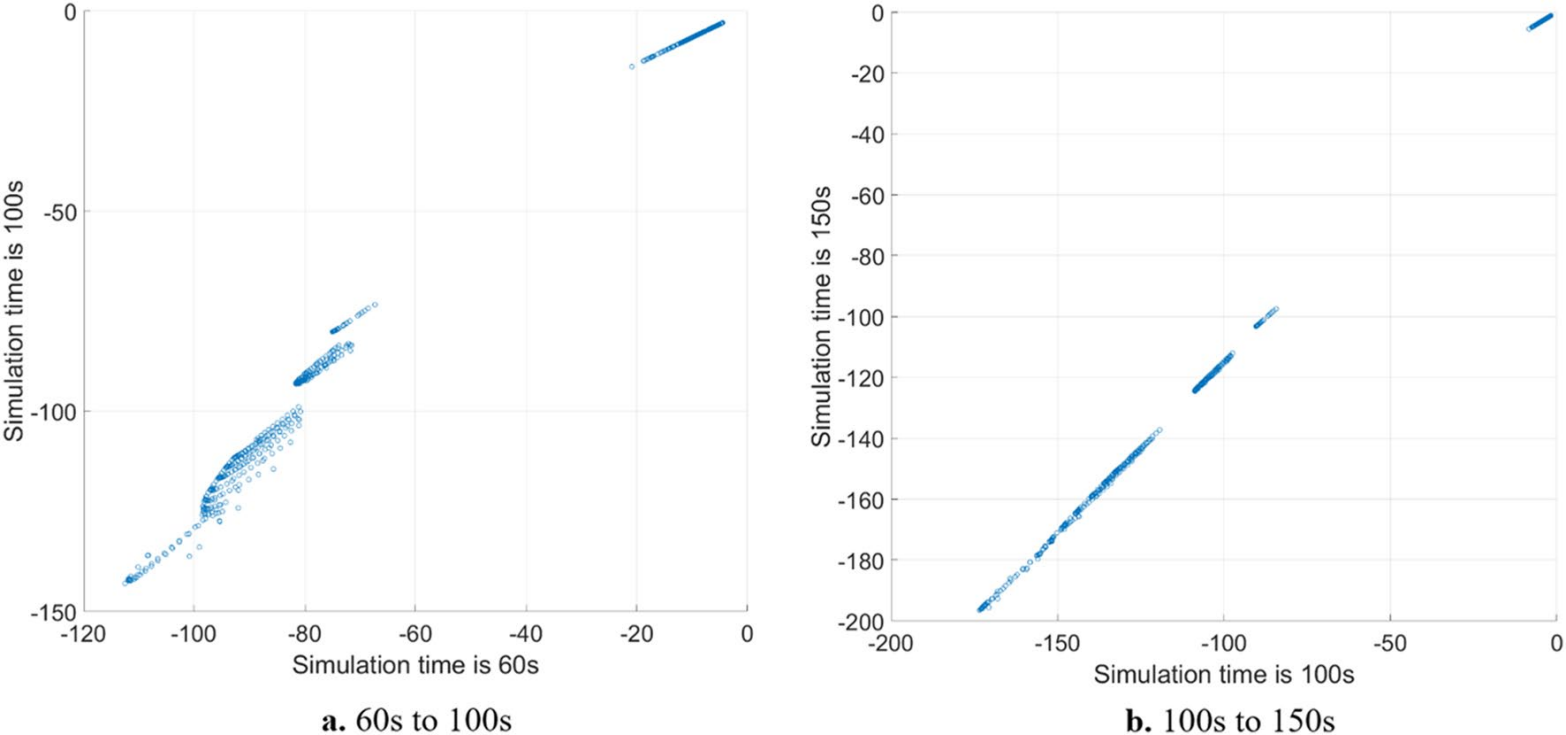
Mapping relation of sum of Lyapunov exponents at different times.

It is significant to set reasonable simulation time and simulation step length for numerical calculation of Lyapunov exponents. In theory, the time should be infinite. But in practical engineering application, enough time should be set to ensure the calculation results of Lyapunov exponents can converge. Meanwhile, the step length should be reasonable to ensure the accuracy of the algorithm. Taking the 5-DOF model as an example, the phase trajectory tends to converge to stable equilibrium point or stable curve after a certain time^25,29^. Because the word number limitation, for more detailed discussion of vehicle system phase trajectory convergence properties, please refer to VSD-2018^29^ and VSD-2021^18^. Corresponding to formula (9), the numerator approximately remains constant and the denominator increases with time. After a certain time, with the increase of time, although the value of Lyapunov exponent change, the relative value between different initial points will not change. In order to better explain this problem and select the appropriate simulation time, it is of necessity to compare the results under different simulation time. 1270 initial points are selected in ${v}_{y}-\omega$ plane. The initial longitudinal velocity is selected as 40 m/s. The initial lateral velocity ranges from − 20 to 20 m/s, and the interval is 1 m/s. The yaw rate is changed from − 1.5 to 1.5 rad/s, and the interval is 0.1 rad/s. Taking low adhesion road and ${\delta }_{f}=0 rad$ as an example, Fig. 3 shows the sum of Lyapunov exponents at different simulation time. It can be seen that the sum of Lyapunov exponents at different time has obvious layer phenomenon. Figure 4 shows the mapping relation of the sum of Lyapunov exponents at different time. When the time changes from 60 to 100 s, as shown in Fig. 4a, it is obvious that the mapping relation is approximately linear, but the linear relation is not perfect, which means that the relative value between different initial points is time-varying. When the time varies from 100 to 150 s in Fig. 4b, the relative value shows a good linearity, which means after 100 s, the relative value and mapping relation between different initial points will be linear over time. Based on the above simulation and references^16^, 100 s is adopted as simulation time for numerical algorithm, and the step is 0.01 s, which is enough for practical engineering application to technically reveal vehicle system global characteristics. It is also reasonable to approach this engineering problem from other perspectives. In addition, the multiplicity of exponents is also an important content, which has been discussed in paper-2016^30^, can help researchers understand the Lyapunov exponents.

## Analysis for global characteristics of 2, 3, 5-DOF models

Based on the VSD-2013^16^, JAUTO-2021^17^ and VSD-2021^18^, this paper further studies quantitative indicator of vehicle stability and dynamic global characteristics. In this section, on the one hand, the global dynamic characteristics of different degrees of freedom models are quantitatively revealed by using Lyapunov exponents; on the other hand, it also reveals the different characteristics of Lyapunov exponents in different degrees of freedom models.

### Global characteristics in 2-DOF model

First, Lyapunov exponents are used to reveal the global characteristics of the 2-DOF vehicle model. The initial longitudinal velocity is 30 m/s. The initial lateral velocity changes from − 10 to 10 m/s. The initial yaw rate changes from − 1 to 1 rad/s. Front wheel steering angle is 0 rad. The simulation time is 100 s.A total of 4131 initial points. High adhesion and low adhesion road condition are calculated respectively.

**Figure 5:**
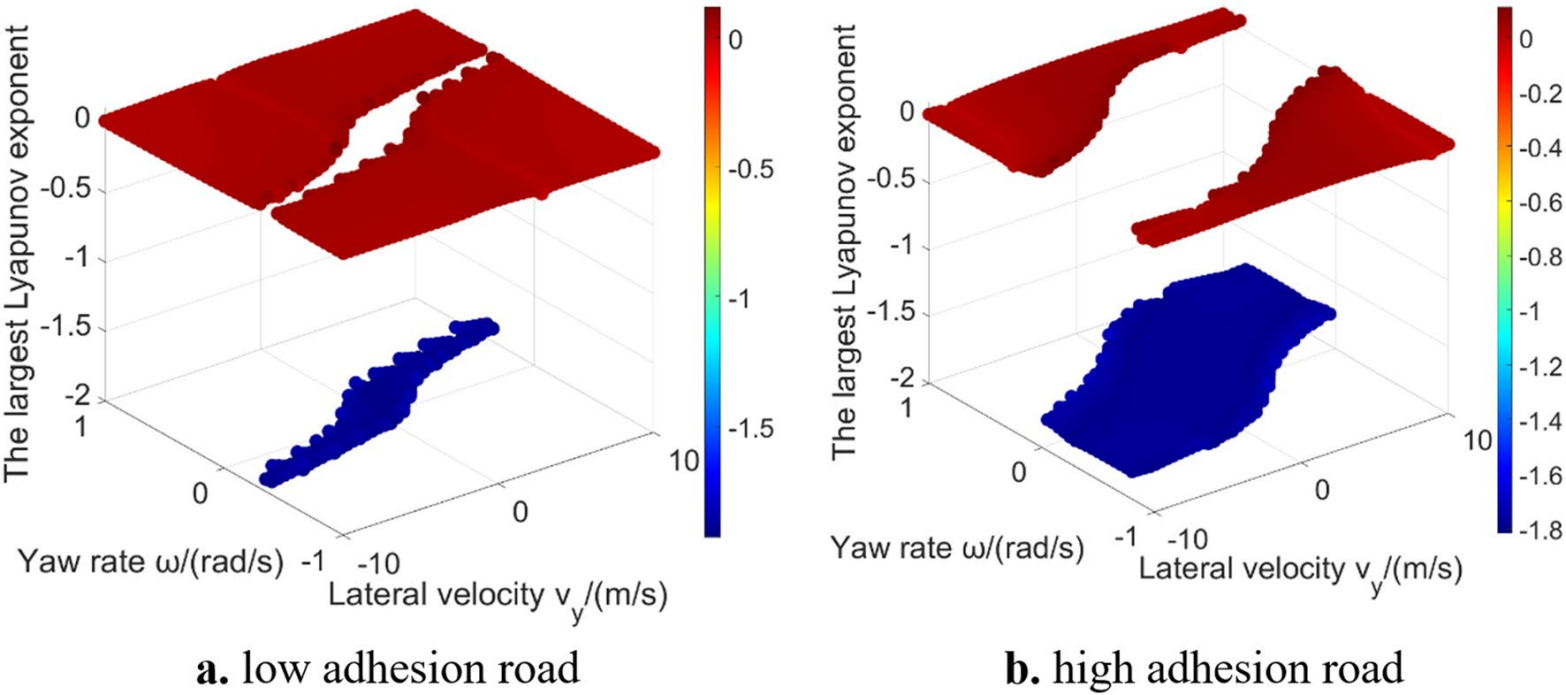
The largest Lyapunov exponent in 2-DOF model global region.

**Figure 6:**
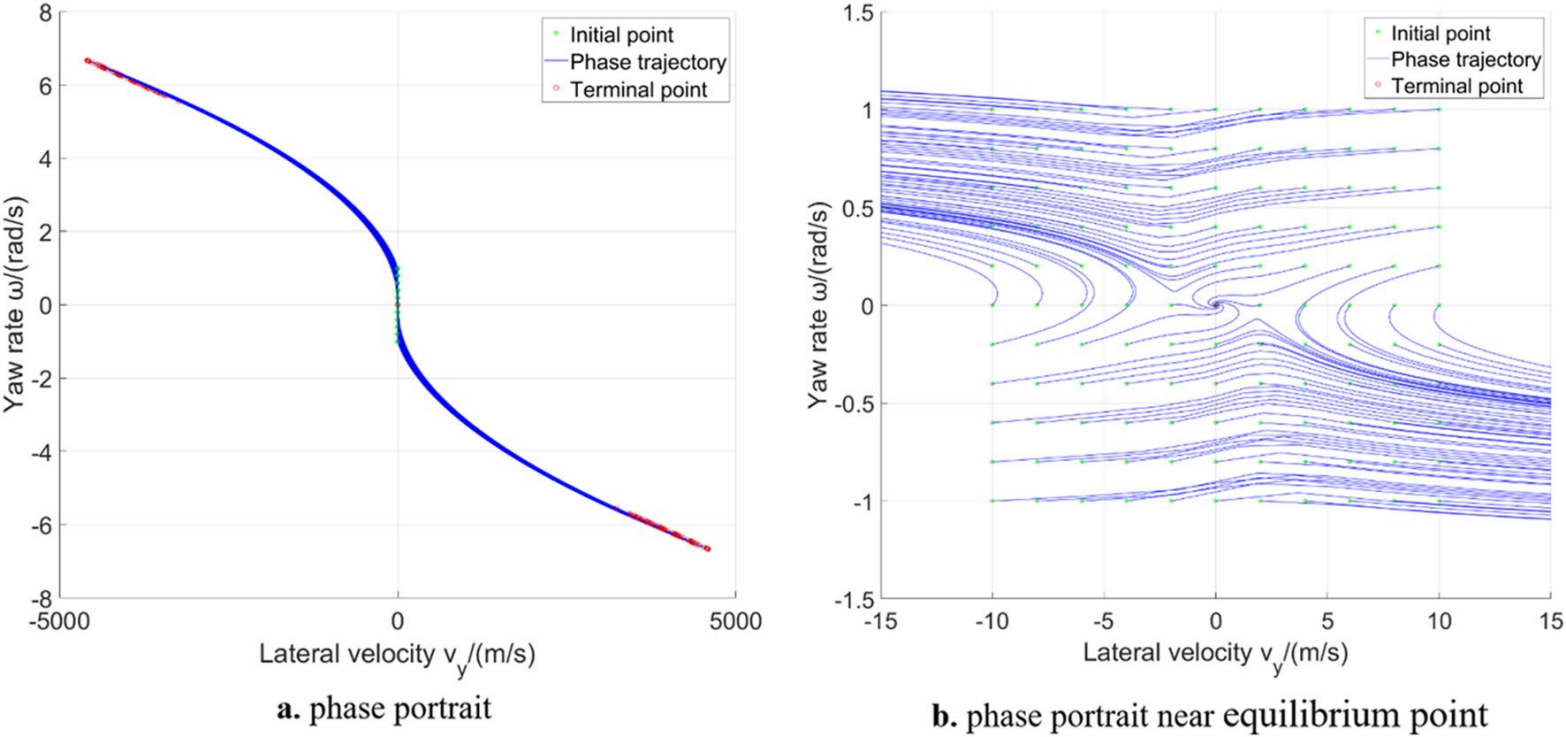
Phase portrait of 2-DOF vehicle system.

Figure 5 shows the results of the largest Lyapunov exponent in global region. The largest Lyapunov exponent in unstable region is positive, which means that the corresponding phase trajectories diverge; while the largest Lyapunov exponent in stable region is negative, which means that the corresponding phase trajectories converge. Figure 6 shows the phase trajectories of the 2-DOF model in ${v}_{x}=30\mathrm{m}/\mathrm{s}$ plane. Figure 6a shows phase trajectories in large range. Figure 6b shows the phase trajectories near the stable equilibrium point. It can be seen from Fig. 6b that some phase trajectories converge to the equilibrium point (${v}_{y}=0$, $\omega =0$). Based on the conclusion of phase plane method, these phase trajectories are located in the stable region. The other phase trajectories are far away from the stable equilibrium point, and the yaw rate and lateral velocity will diverge to infinity over time, belonging to the unstable region. This is why the stable region and the unstable region of 2-DOF model can be easily solved by using Lyapunov exponents. However, owning to constant longitudinal velocity, 2-DOF model introduces strong limitations and cannot accurately reflect the dynamic characteristics in unstable region^14^. The differences between 2-DOF model and 3-DOF model will be further discussed in 4.2 section, to supplement the conclusions obtained from the 2-DOF model.

**Figure 7:**
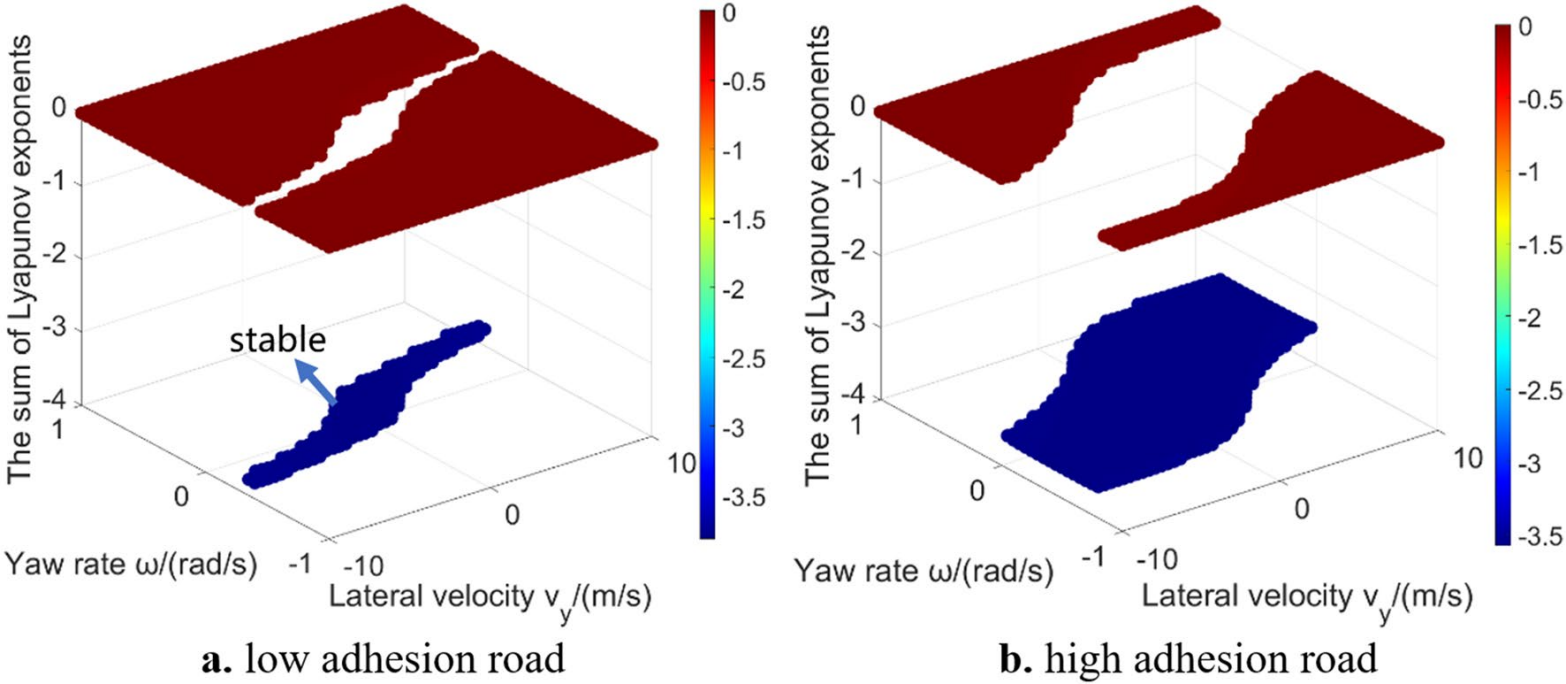
The sum of Lyapunov exponents in 2-DOF model global region.

In addition to the largest Lyapunov exponent, the sum of Lyapunov exponents is also an important indicator to quantitatively reveal the dynamic characteristics of the system, which reflects the time-averaged divergence of the phase space velocity^27^. Figure 7 shows the sum of Lyapunov exponents. Both the largest Lyapunov exponent and the sum of Lyapunov exponents can easily reveal the stable region and unstable region of the 2-DOF vehicle system by quantitative value. Wu et al.^16^ also used this global characteristic to solve the stable region of 2-DOF vehicle system. However, current researches mainly stay on the 2-DOF models, and don’t explain whether this analysis method is applicable to high-DOF models. The characteristics of Lyapunov exponents in high-DOF models global region also need be further analysed. In addition, estimation of the sum of Lyapunov exponents is redundant for 2-DOF vehicle system. According to the Poincaré–Bendixson theorem, the only possible attractors in such system are fixed points and limit cycles^31^. Therefore, if the largest Lyapunov exponent is positive, then the system is unstable and trajectory escapes to infinity.

### Global characteristics in 3-DOF model

**Figure 8:**
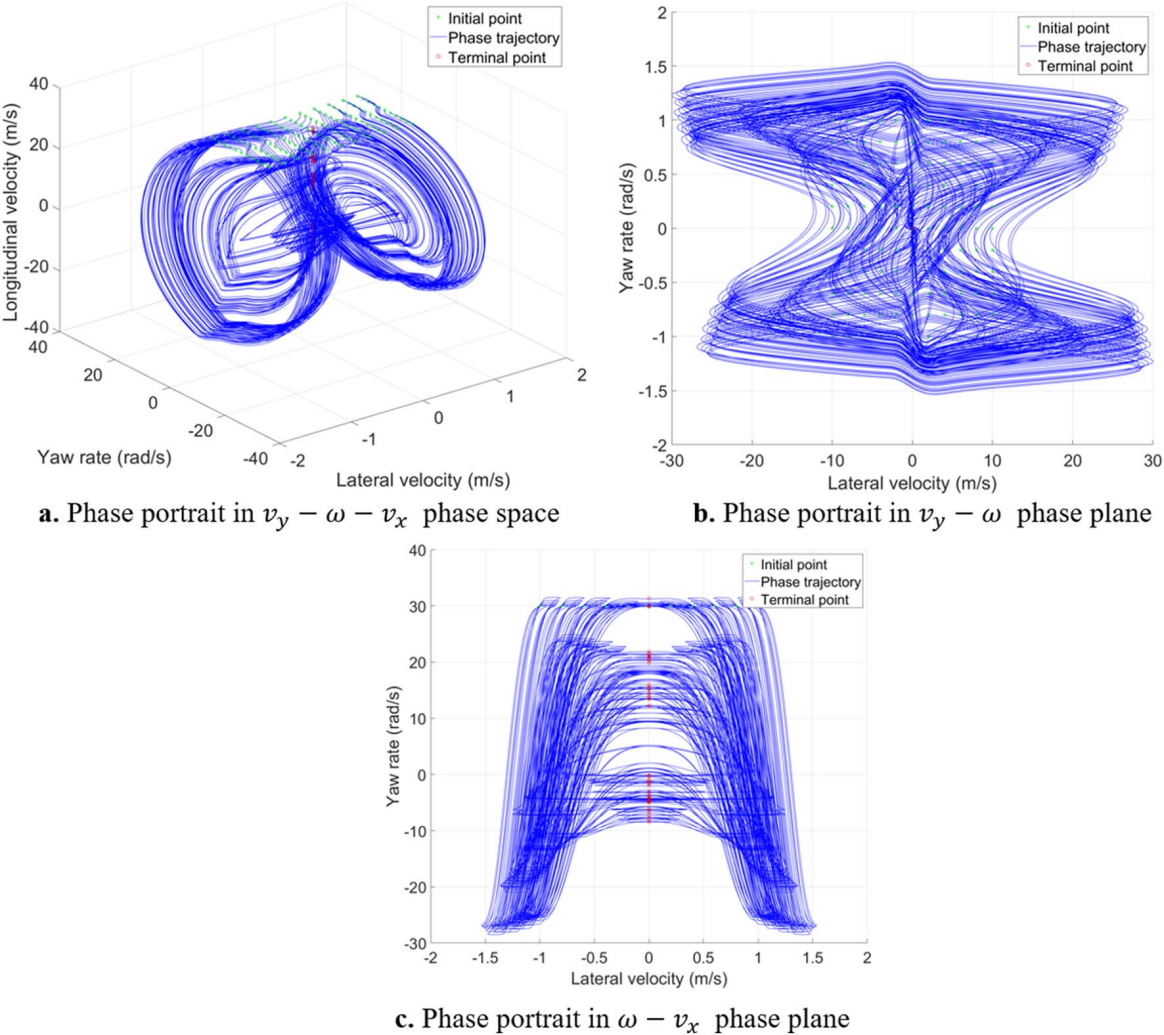
Phase portrait of 3-DOF vehicle system.

The 3-DOF system introduces the longitudinal velocity change, which can more realistically reflect the dynamic characteristics in global region. The phase portrait of the 3-DOF model with ${\delta }_{f}=0\mathrm{ rad}$ in ${v}_{x}=30\mathrm{m}/\mathrm{s}$ plane is shown in Fig. 8. It can be seen that the 3- DOF model does not have the problem that the states extend to infinity over time, which solves the shortcoming of the 2-DOF model. In the case of ${\delta }_{f}=0 \mathrm{rad}$, the phase trajectories of the 3-DOF model will converge to the straight line at (${v}_{y}=0$,$\omega =0$)^25^, but the longitudinal velocities of the equilibrium points are different. In addition, it can also be seen that when using the phase plane method to analyse the high-DOF models, the phase trajectories are much more cluttered than 2-DOF model, which is not easy to analyse.

**Figure 9:**
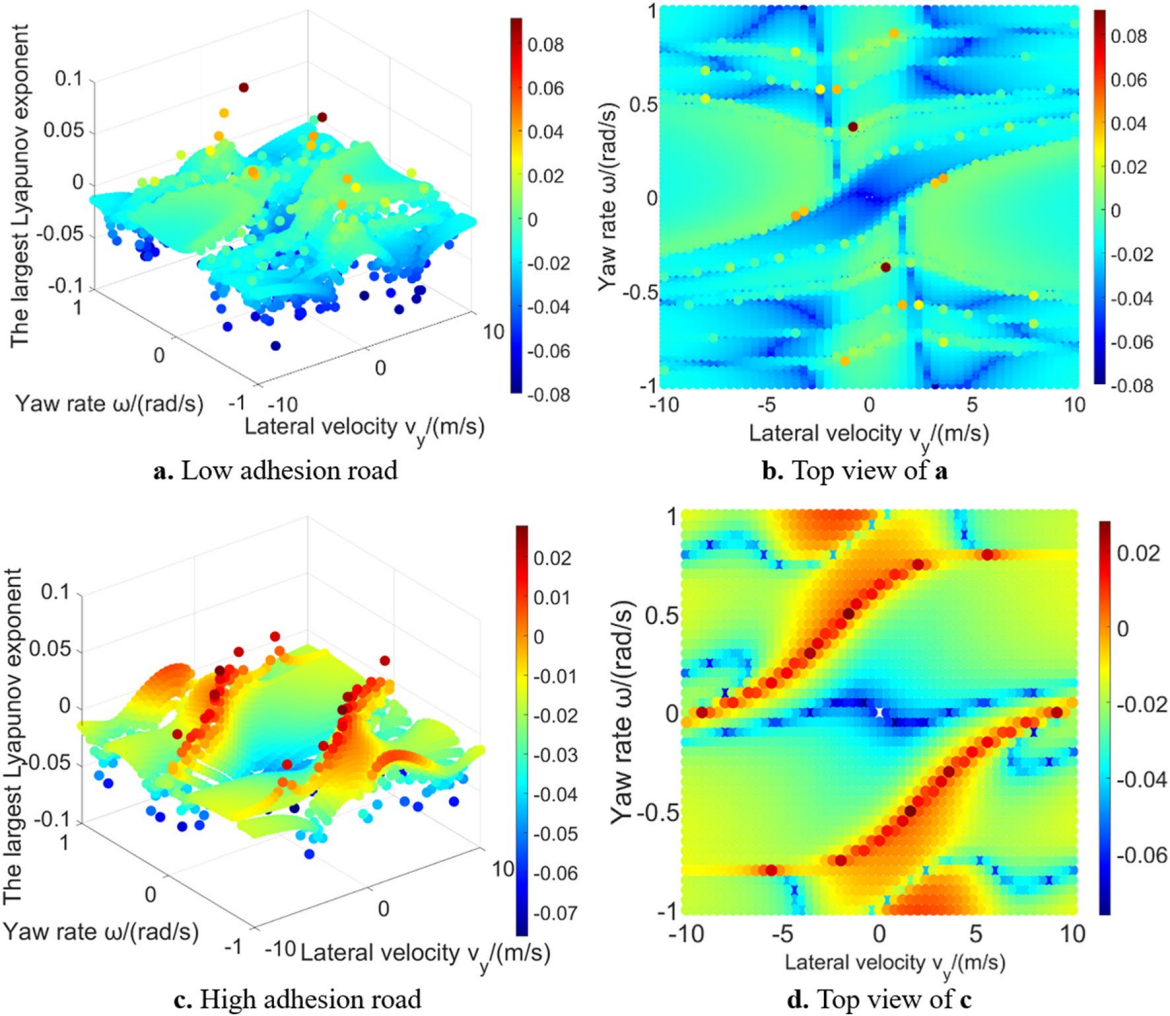
The largest Lyapunov exponent in the 3-DOF model global region.

Figure 9 shows the largest Lyapunov exponent in global region under low and high adhesion road conditions with the longitudinal velocity is 30 m/s. It can be seen that the results of 3-DOF model is significantly different from 2-DOF model because the longitudinal velocity is introduced, which increases the complexity of the system. It can be vaguely seen that there is a shape of stable region in the middle region, but it cannot clearly distinguish stable region and unstable region according to the value of the largest Lyapunov exponent, which supplements the research conclusion of Wu et al.^16^. Moreover, as the complexity of the system increases, the difficulty of calculation also increases, and there are outliers caused by numerical calculation problems. Most outliers are on the boundary of the vehicle system layered region^18^, which may be caused by the selected numerical method of Lyapunov exponents estimation.

**Figure 10:**
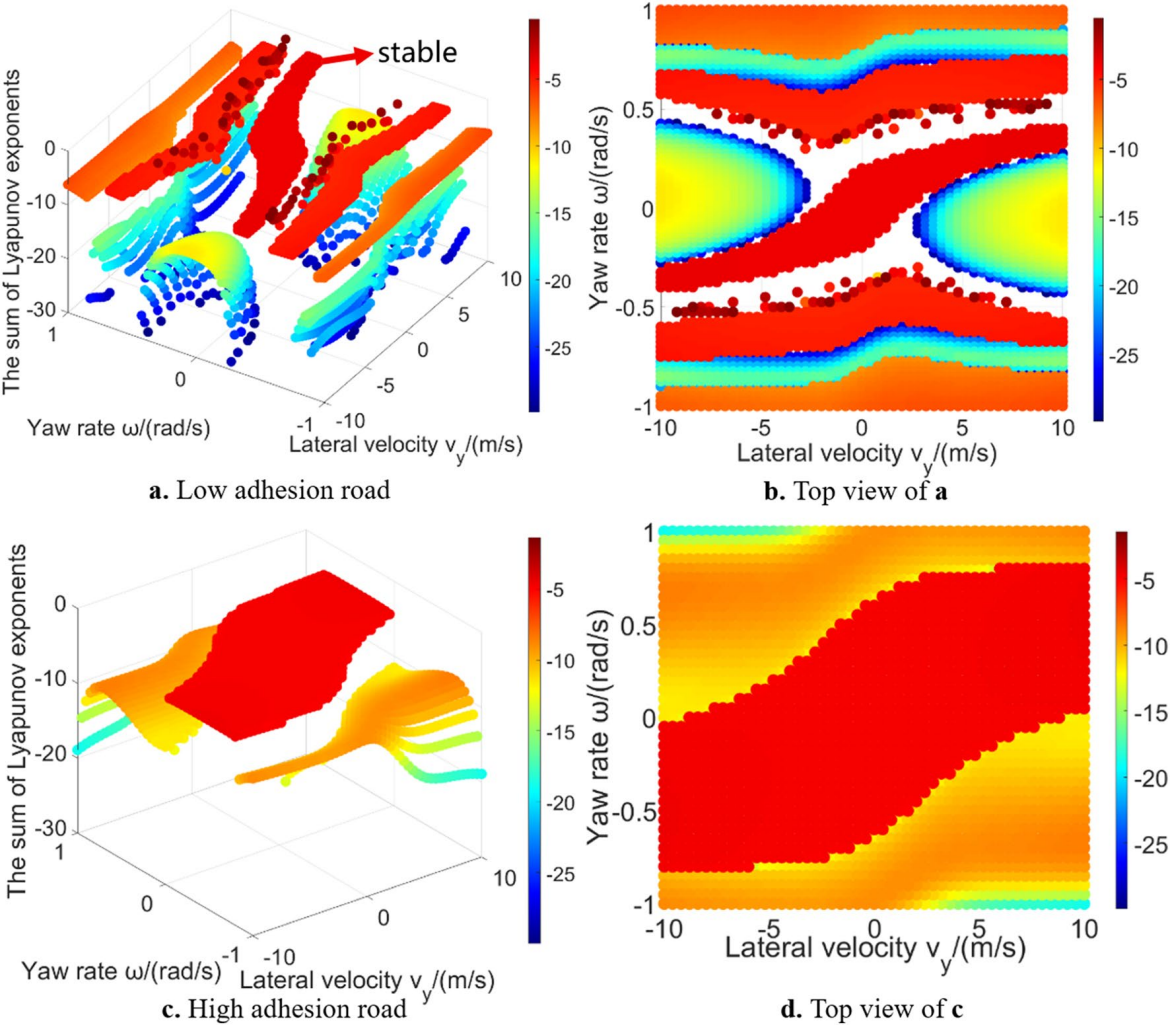
The sum of Lyapunov exponents in 3-DOF model global region.

**Figure 11:**
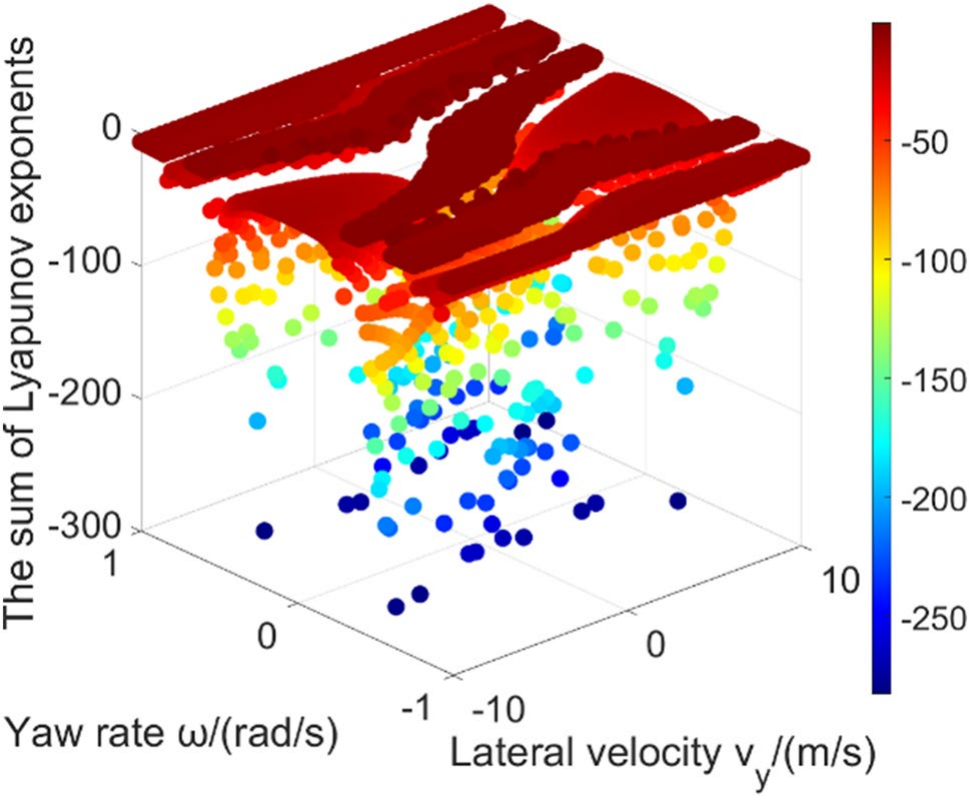
Sum of Lyapunov exponents (> − 300) in the global region on low adhesion road.

Figure 10 gives the sum of Lyapunov exponents in global region. These data points’ value is larger than − 30. The purpose is to better show the relative difference in those regions (Fig. 11 gives the data points whose value is larger than − 300 in low adhesion road. It can be seen that the calculation result in some region is far less than − 30, which affects the presentation effect). The calculation result of sum of Lyapunov exponents is related to the convergence process of the phase trajectories. In the 2-DOF model, all initial states either converge to a unique equilibrium point or extend to infinity. However, in the 3-DOF model, all phase trajectories will finally converge without diverging to infinity^18,29^. The result of largest Lyapunov exponent is around zero. Result in the vehicle stable region tends to be slightly less than zero. Result is zero meaning that the phase space has a varying direction that neither exponentially diverges nor converges. Because the longitudinal velocity of the equilibrium points which phase trajectories converge to is different (as shown in Fig. 8): in stable region, the longitudinal velocity which the phase trajectories converge to is relatively high, thus the final longitudinal velocity has not changed much compared with the initial longitudinal velocity; the phase trajectories in 3-DOF vehicle unstable region finally converges to a lower longitudinal velocity, thus the final longitudinal velocity decreases a lot compared with the initial longitudinal velocity. When using Lyapunov exponents to calculate the convergence of phase trajectories, the sum of Lyapunov exponents in stable region is larger than that in unstable region, because the 3-DOF model considers the convergence in the longitudinal velocity direction. In addition, there are obvious outliers on the boundary of stable region, which also indicates that the calculation difficulty of Lyapunov exponent method will significantly increase as the system degrees of freedom increasing.

The value and regional characteristics of Lyapunov exponents in 3-DOF model are different from that in 2-DOF model. In 2-DOF model unstable region, because all the phase trajectories tend to infinity, thus the Lyapunov exponents have no obvious layer characteristic. In 3-DOF model, because the phase trajectories converge to different equilibrium points, Lyapunov exponents reveal layer characteristic different from 2-DOF. In our previous work^18^, this characteristic was called layer phenomenon, which was more obvious on low adhesion road. Because the main purpose of this paper is to analyse the Lyapunov exponents, please refer to our previous work^18^ for detailed reason of such layer characteristic.

From the above analysis, it can be seen that the global characteristics in high-DOF vehicle model are different from 2-DOF model, like global value characteristic and global region characteristic. This is what needs to be paid attention to in research of the Lyapunov exponents method, which supplements the previous research conclusions by Wu, Shi et al.^16,17^.

### Global characteristics in 5-DOF model

**Figure 12:**
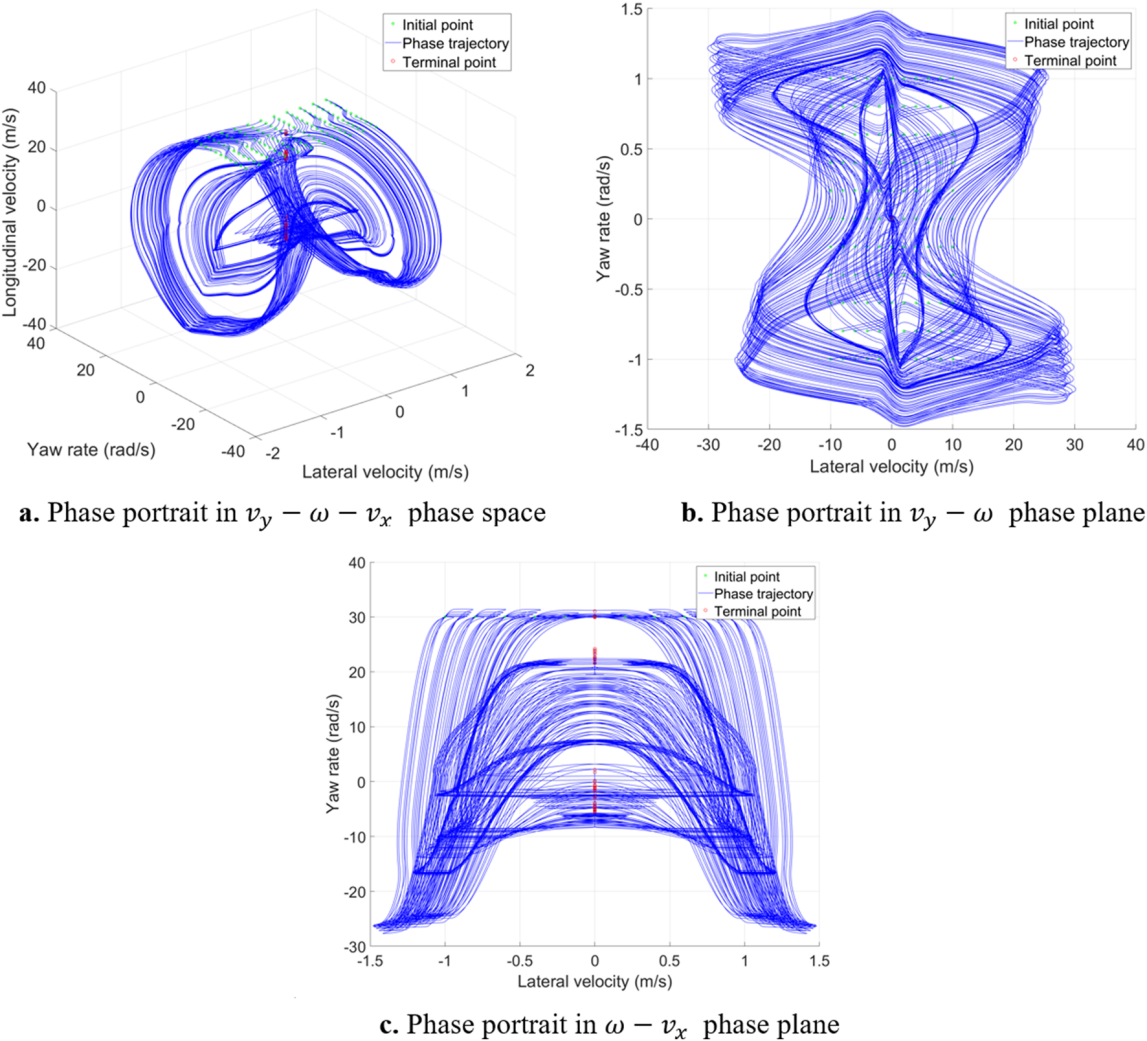
Phase portrait of 5-DOF vehicle system.

**Figure 13:**
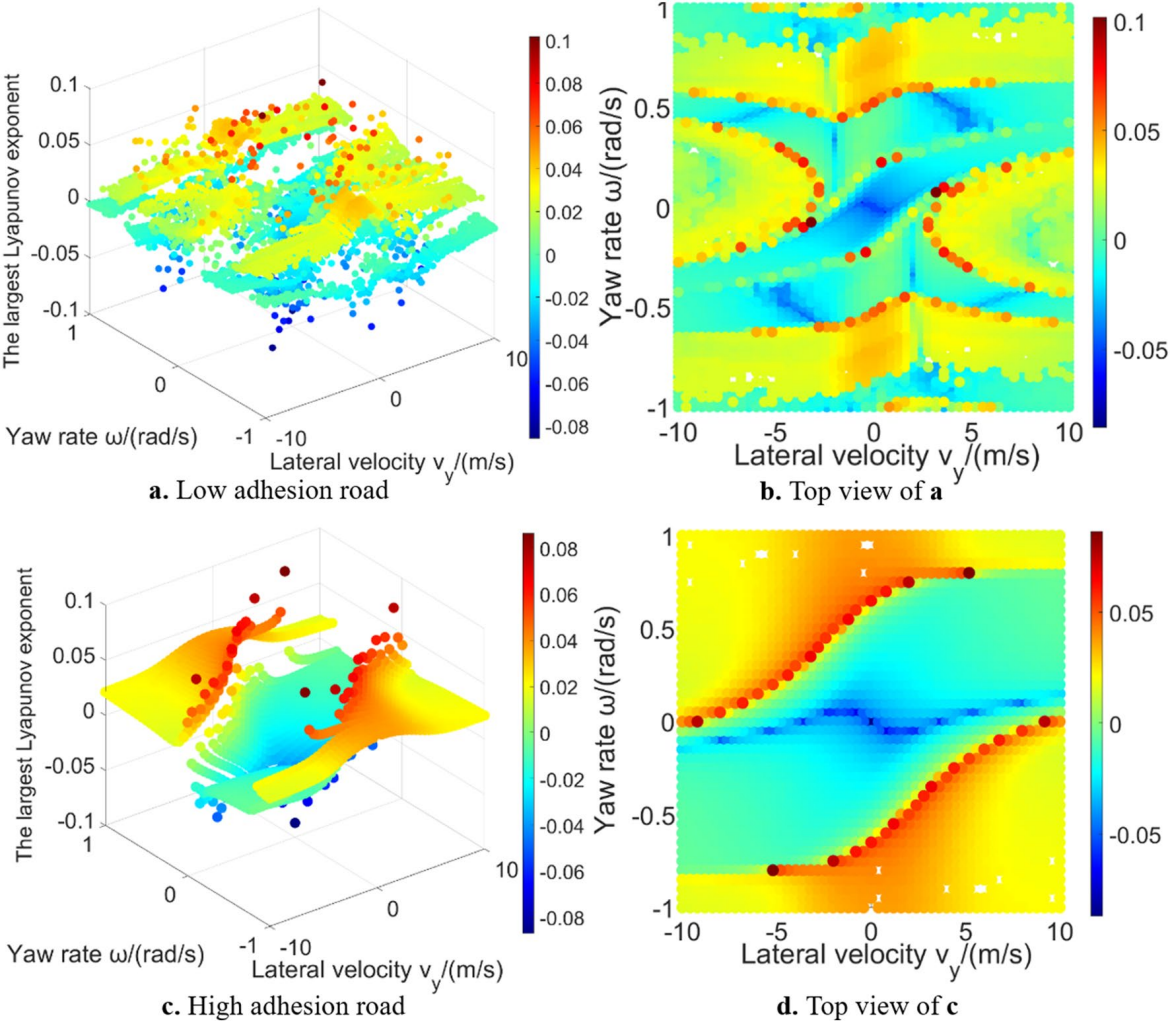
The largest Lyapunov exponent in the 5-DOF model global region.

As vehicle model becomes more complex, 5-DOF model introduces two degrees of freedom for front and rear tire rotation, whose phase trajectories in ${v}_{x}=30\mathrm{m}/\mathrm{s}$ initial points plane is similar to that in 3-DOF model, as shown in Fig. 12. The ${T}_{df}$ and ${T}_{dr}$ is $0 \mathrm{N}\cdot \mathrm{m}$. The ${\delta }_{f}=0 \mathrm{rad}$. Figure 13 shows the largest Lyapunov exponent in global region in 5-DOF vehicle model. It is almost consistent with the conclusion obtained from analysis of the 3-DOF model. The shape of the stable region can only be vaguely seen in the middle region, which cannot clearly distinguish the stable region from the unstable region according to the value. Therefore, it is necessary to further analyse the sum of Lyapunov exponents. In addition, due to the increase of model complexity, the calculation becomes more difficult, leading to calculation failure at some initial points, which have been removed in the figure. Computation times for Lyapunov exponents for the 2-DOF vehicle system is about 8 s; 3-DOF is about 25 s; 5-DOF is about 55 s.

**Figure 14:**
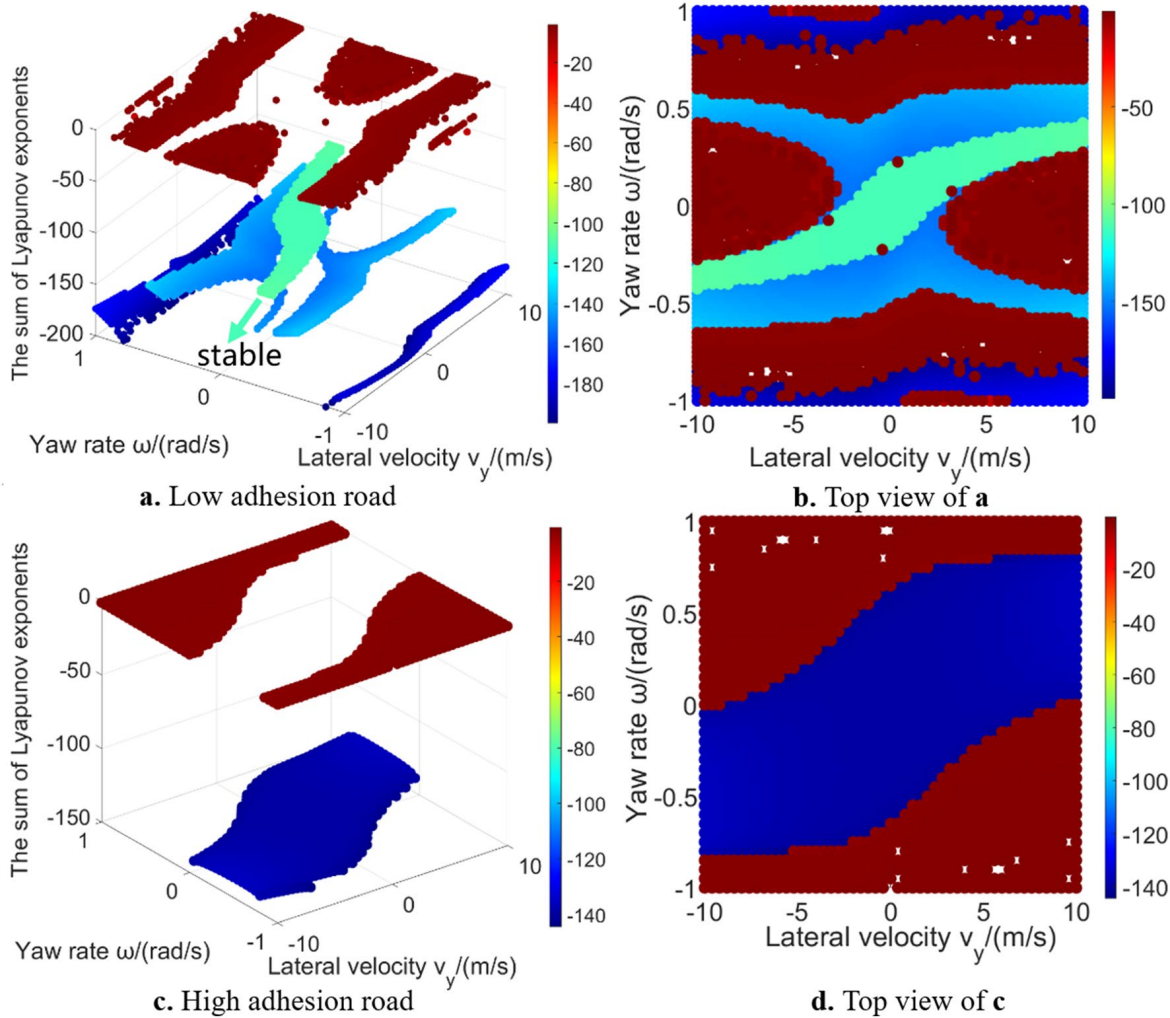
The sum of Lyapunov exponents in 5-DOF model global region.

Figure 14 shows the solution results of the sum of Lyapunov exponents in the global region of the 5-DOF vehicle model. Due to the introduction of two new degrees of freedom, the relative value characteristics between stable and unstable region is different from that of 2-DOF and 3-DOF models. Taking low adhesion road as an example, in the 2-DOF model, the value in stable region is smaller than that in unstable region; in the 3-DOF model, the value in stable region is larger than that in unstable region; in the 5-DOF model, the value in stable region is smaller than that in a part of unstable region, but larger than other part. In orthogonalization algorithm, the changes of the five variables in the 5-DOF model are considered equally. However, in practical, the longitudinal velocity, lateral velocity and yaw rate are more important than the rotational angular velocity of front and rear wheels. Thus, when using the Lyapunov exponents method to analysis vehicle system global characteristics, more complicated model leads to different relative value characteristic, although the introduced new degrees of freedom or variables are not very important to reveal the vehicle dynamic characteristics. Therefore, considering more degrees of freedom will influence the relative value characteristic of Lyapunov exponents. Although the sum of Lyapunov exponents of different degrees of freedom models has different value characteristic, they all show the layer characteristic. The sum of Lyapunov exponents can also quantitatively reveal layer phenomenon^18^ to distinguish stable region and unstable region in 5-DOF vehicle system.

## Conclusions and future work

At present, there are two significant research directions in the field of vehicle dynamics. The first is to research the global dynamics characteristics, such as vehicle drift, collision avoidance when vehicle is unstable, global region characteristics, etc. The second essential direction is to research whether the conclusions drawn from low-DOF models (most from 2-DOF) can be successfully extended to nonlinear high-DOF models. In the current work, researchers considered Lyapunov exponent as a quantitative indicator and used it in nonlinear vehicle system dynamic analysis, but lack of the analyse of global characteristics in high-DOF model. Aiming at this key problem, the global characteristics of Lyapunov exponents under different DOF models are compared comprehensively in this paper. It is explained that the relative value characteristic of Lyapunov exponents will change due to the introduction of new degrees of freedom into the high degrees of freedom model. Moreover, Lyapunov exponents can reveal the region characteristic in different degrees of freedom models. For 3-DOF and 5-DOF models, the sum of Lyapunov exponents shows better performance for layer characteristic than the largest Lyapunov exponent. Through the above work, this paper quantitatively reveals the global characteristics of Lyapunov exponents in the nonlinear vehicle plane motion system, which supplement the relevant conclusions. In future work, it is valuable to compare the Lyapunov exponents with other quantitative indicators, such as dissipation of energy, side slip angle, convergence time and so on.

## Data Availability

Due to space limitation, this paper only shows partial results. The datasets generated during and/or analysed during the current study are available from the corresponding author on reasonable request.

## References

[1] Abe M (2015). Vehicle Handling Dynamics: Theory and Application.

[2] Karnopp D (2004). Vehicle Stability.

[3] Mitschke M, Wallentowitz H (1972). Dynamik der Kraftfahrzeuge.

[4] Inagaki S, Kushiro I, Yamamoto M (1995). Analysis on vehicle stability in critical cornering using phase-plane method. JSAE Rev..

[5] Yamamoto M, Koibuchi K, Fukada Y, Inagaki S (1996). Vehicle stability control in limit cornering by active brake. Trans. Soc. Automotive Eng. Japan.

[6] Ono E, Hosoe S, Tuan HD, Doi SI (1998). Bifurcation in vehicle dynamics and robust front wheel steering control. IEEE Trans. Control Syst. Technol..

[7] Catino, B., Santini, S. & Di Bernardo, M. in *42nd IEEE International Conference on Decision and Control.* 2252–2257 (IEEE).

[8] Nguyen, V. *Vehicle handling, stability, and bifurcaiton analysis for nonlinear vehicle models*, MSc Thesis, University of Maryland, College Park, Maryland, USA, (2005).

[9] Shen S, Wang J, Shi P, Premier G (2007). Nonlinear dynamics and stability analysis of vehicle plane motions. Veh. Syst. Dyn..

[10] Horiuchi S (2012). Evaluation of chassis control method through optimisation-based controllability region computation. Veh. Syst. Dyn..

[11] Horiuchi S, Okada K, Nohtomi S (2008). Analysis of accelerating and braking stability using constrained bifurcation and continuation methods. Veh. Syst. Dyn..

[12] Ko YE, Lee JM (2002). Estimation of the stability region of a vehicle in plane motion using a topological approach. Int. J. Veh. Des..

[13] Ko Y, Song C (2010). Vehicle modeling with nonlinear tires for vehicle stability analysis. Int. J. Automot. Technol..

[14] Liu L, Shi S, Shen S, Chu J (2010). Vehicle planar motion stability study for tyres working in extremely nonlinear region. Chin. J. Mech. Eng..

[15] Genesio R, Tartaglia M, Vicino A (1985). On the estimation of asymptotic stability regions: State of the art and new proposals. IEEE Trans. Autom. Control.

[16] Sadri S, Wu C (2013). Stability analysis of a nonlinear vehicle model in plane motion using the concept of Lyapunov exponents. Veh. Syst. Dyn..

[17] Shi S, Meng F, Bai M, Lin N (2021). The stability analysis using Lyapunov exponents for high-DOF nonlinear vehicle plane motion. Proc. Inst. Mech. Eng. Part D-J. Automob. Eng..

[18] Meng F (2021). Dissipation of energy analysis approach for vehicle plane motion stability. Veh. Syst. Dyn..

[19] Tamer A, Masarati P (2016). Stability of nonlinear, time-dependent rotorcraft systems using lyapunov characteristic exponents. J. Am. Helicop. Soc..

[20] Masarati P, Tamer A (2015). Sensitivity of trajectory stability estimated by Lyapunov characteristic exponents. Aerosp. Sci. Technol..

[21] Tamer A, Masarati P (2019). Sensitivity of lyapunov exponents in design optimization of nonlinear dampers. J. Comput. Nonlinear Dyn..

[22] Masarati P (2013). Estimation of Lyapunov exponents from multibody dynamics in differential-algebraic form. Proc. Inst. Mech. Eng. Part K J. Multi-body Dyn..

[23] Pacejka H (2005). Tire and Vehicle Dynamics.

[24] Pacejka HB, Bakker E (1992). The magic formula tyre model. Veh. Syst. Dyn..

[25] Mu Y, Li L, Shi S (2018). Modified tire-slip-angle model for chaotic vehicle steering motion. Automot. Innov..

[26] Yang C, Wu Q (2010). On stability analysis via Lyapunov exponents calculated from a time series using nonlinear mapping—a case study. Nonlinear Dyn..

[27] Wolf A, Swift JB, Swinney HL, Vastano JA (1985). Determining Lyapunov exponents from a time series. Phys. D.

[28] Ramasubramanian K, Sriram MS (2000). A comparative study of computation of Lyapunov spectra with different algorithms. Phys. D Nonlinear Phenomena.

[29] Beal CE, Boyd C (2019). Coupled lateral-longitudinal vehicle dynamics and control design with three-dimensional state portraits. Veh. Syst. Dyn..

[30] Masarati P, Tamer A (2016). The real schur decomposition estimates lyapunov characteristic exponents with multiplicity greater than one. Proc. Inst. Mech. Eng. Part K J. Multi-body Dyn..

[31] Strogatz SH (2018). Nonlinear Dynamics and Chaos: With Applications to Physics, Biology, Chemistry, and Engineering.

